# 

**Published:** 1896-07

**Authors:** 


### BOOKS RECEIVED.

A Text-book of Bacteriology. By George M. Sternberg, M. D., LL.D., Surgeon-General U. S. Army, ex-President American Public Health Association, etc., 8vo, pp. 693. Illustrated by Heliotype and Chromolithographic plates and 200 engravings. Price, $5.00. New York: William Wood & Company. 1896.

The Diagnosis and Treatment of Diseases of the Rectum, being a practical treatise on fistula, piles, fissure and painful ulcer, procidentia, polypus, stricture, cancer, etc. By William Allingham, F. R. C. S., England; ex-Member of Council of the Royal College of Surgeons of England; late Senior Surgeon to St. Mark's Hospital for Diseases of the Rectum, etc., and Herbert W. Allingham, F. R. C. S., England, Surgeon to the Great Northern Hospital, etc., 8vo, pp. xvi—485. Sixth edition. New York: William Wood & Company. 1896.

A Manual of Anatomy. By Irving S. Haynes, Ph. B., M. D., Adjunct Professor and Demonstrator of Anatomy in the Medical Department of the New York University; Visiting Surgeon to the Harlem Hospital, etc., 8vo, pp. 680, with 134 half-tone illustrations and 42 diagrams. Price, $2.50 net. Philadelphia: W. B. Saunders, 925 Walnut street. 1896.

Borderland Studies. Miscellaneous Addresses and Essays pertaining to Medicine and the Medical Profession, and their Relations to General Science and Thought. By George M. Gould, A. M., M. D., formerly editor of the Medical News. Price, $2.00. Philadelphia : P. Blakiston, Son & Co., 1012 Walnut street. 1896.

How to Feed Children. A Manual for Mothers, Nurses and Physicians. By Louise E. Hogan. Price, $1.00. Philadelphia : J. B. Lippincott Company. 1896.

A Compend of Gynecology. By William H. Wells, M. D., Adjunct Professor of Obstetrics and Diseases of Infancy in the Philadelphia Polyclinic, etc. Quiz Compends, No. 7. Duodecimo, with 150 illustrations. Price, 80 cents. Philadelphia : P. Blakiston, Son & Co., 1012 Walnut street. 1896.

A Compend of Diseases of Children. Especially adapted for the use of Medical Students. By Marcus P. Hatfield, A. M., M. D., Professor of Diseases of Children, N. W. U. Medical School, etc. Quiz Compends, No. 14. Duodecimo, second edition, thoroughly revised. Price, 80 cents. Philadelphia : P. Blakiston, Son & Co., 1012 Walnut street. 1896.

Merck's 1896 Index. An Encyclopedia for the Physician and Pharmacist. Price, $5.00. Merck & Co., New York.

The Three Ethical Codes. That of the American Medical Association ; Its Constitution, By-laws, Amendments, etc. That of the American Institute of Homeopathy and that of the National Eclectic Medical Society. Limp cloth, round corners, 55 pages, postpaid 50 cents. The Illustrated Medical Journal Co., Publishers, Detroit, Mich.

Medical and Surgical Report of the Presbyterian Hospital in the City of New York. Volume I., January, 1896. Edited by Andrew J. McCosh, M. D., and Walter B. James, M. D. New York : The Knickerbocker Press.

### Literary Notes.

### INDEX TO VOLUME XXXV.

PAGE.

ABORTION, Early Treatment of . . . . . . . . . . . . 173
Abortions, Indications for Induction of . . . . . . . . . . . . 252
Abbott, A. C., M. D., Principles of Preventive Inoculation and Serum Therapeutics . . . . . . . . 609
Adenoid Vegetations . . . . . 722
Adirondacks as a Winter Health Resort . . . . . . . . . . . . . . 385
Albuminuria . . . . . . . . . . . . 158
Alcoholism, Treatment of . . . . . 217
Alcohol in Fevers . . . . . . . . . 415
Ambulance Collision . . . . . . . . 270
Ambulances, Electric Railway . . . 508
American Students in France . . . 510
Medical Association, Semicentennial Celebration of . . . . . . . . . . . 825
Amputation, Synchronous . . . . . 50
Amputations, New Method in Thigh. 892
Anemia, Pernicious . . . . . . . . . 811
Antiseptics in Surgery . . . . . . . 224
Anti-noise League . . . . . . . . . 509
Ani Pruritus . . . . . . . . . . . . 575
Appendicitis without Preceding Pain or Fever . . . . . 469
Classification of . . . . . . 579
Illustrative Cases of . . . 537
Army Medical Examinations . . . 968
Asafetida in Obstetrics . . . . . . 818
Ataxia, Locomotor . . . . . . 243, 246
Atropia, Abuse and Use of in Eye Treatment . . . . . . . . . . . 149

BACKACHE from Weak Heart . 29
Baker, C. O., M. D., Localised Peritonitis . . . . . . . . . 559
Baths, the People's . . . . . . . . 352
Bartlett, F. W., M. D., Treatment of Diphtheria . . . . . . . . . . 647
Benninghoff, G. E., M. D., Medical Surgery . . . . . . . . . . . . . 710
Benzosol, Therapeutic Value of . . 728
Bill, A Dangerous . . . . . . . . . 825
Bissell, William G., M. D., Tubercular Infection in Street Cars . . . . . . . . . . 46
Examination of Sputum . . 260
Experience in Bacteriology of Diphtheria . . . 626

PAGE.

Blepharitis, Formulæ for . . . . . 664
Breast, Remarks on Cancer of . . . 1
Malignant Disease of . . . 898
Breech Labors. The Fillet in . . . 290
Books received . . . . . 202, 286, 363, 444, 522, 604, 684, 764, 845, 926, 991
Buffalo Healthfulness of . . . . . 898
Morning Express . . . . . 589
Railway Company . . . . 422
Burial Casket, Sanitary . . . . . . 63
Burns, Picric Acid for . . . . . . 893

CANCER of the Breast . . . . 1
Cardiac Embolism following Abdominal Section . . . . 48
Carroll, Jane W, M. D, Cystic Urachus . . . . . . . . . 869
Calculus in the Kidney . . . . . . 655
Canaliculus, which to slit . . . . . 650
Cataract, Operation for Secondary . 152
Treatment of Immature . . 807
Catarrh, Nasal . . . . . . . . . . . 492
Censors, Board of Report of Erie County . . . . . . . . . . . . . 966
Cerebral Softening, Case of . . . . 864
Cerumen, Impacted . . . . . . . . 16
Chautauqua County, Notes from . . 179
Chemistry, Rapid Strides in . . . . 745
Chloroform and Ether, First Uses of in Buffalo . . . . . . 689
Administration, Dangers and Treatment . . . . . 695
Christian Scientists, Illegal Practice by . . . . . . . . . . . 594
Scientists and Health Department . . . . . . . . . 670
Chutmucks, The Chief of . . . . . 269
Cigarette Habit . . . . . . . . . . . 724
Clergymen, Attendance of at Cemeteries . . . . . . . . . . . . . 747
Clubfoot, Congenital . . . . . . . 889
and Pyelitis . . . . . . . 653
Code Anomalies . . . . . . . . 510
Coitus, Sudden Death during . . . 253
Colic, Renal in Infancy . . . . . . 896
College Life for Women Twenty Years Ago . . . . . . . . . . . 854
Conklin, William L., M. D., A Pseudencephalic Monster . . . . 638
Cold in the Head . . . . . . . . . 739
Constipation in Women . . . . . . 658

PAGE.
Contemporaries, What ours think of us . . . . . . . . . . . . . 181, 430
Contrexeville, Waters of . . . . . 326
Cornea, Treatment of Wounds of . 806
Coroner, Office of . . . . . . . . . 667

**Correspondence**:

Berlin Letter, Julius Ullman, M. D. . . . . . . . . . . . . 261
Criminal Responsibility, T. D. Crothers, M. D. . . . . . . . . 416
Three Children at one Birth, C. F. Durand, M. D. . . . . . 418
Mydriatics in Refraction, Arthur G. Bennett, M. D. . . . 501
Refraction without Mydriatics, R. H. Satterlee, M. D. . . . 505

Corsets, Advantages of Well-fitting . 915
Craig Colony opening . . . . . . . 591
Crego, F. S., M. D., Treatment of Headache . . . . . . . . . . . 369
Cremation Statistics . . . . . . . . 591
Criminals' Plea of Irresponsibility . 316
Criticism, an Unjust . . . . . . . . 586
Cronyn, John, M. D., Establishment of Niagara University . . . . 780
Croup, Treatment of . . . . . . . 161
Curette, Use and Abuse of . . . . . 658
Cystitis in Children . . . . . . . . . 653

DORR, S. G., M. D., Care and Treatment of Old People . . 144
Doyle, Gregory, M. D., A Broken Neck, Recovery . . . . . . . . . 464
Dunham, Sidney A., M. D., Treatment of Alcoholism . . . . . . . 217

ECTOPIC Gestation, twice in same Patient . . . . . . . . 657
Electricity in Nervous Diseases . . 300
Electrolysis, Linear . . . . . . . . 488
Elsner, S. L., M. D., Appendicitis . 469
Encephalitis, Acute . . . . . . . . 809
Epilepsy, Reflex . . . . . . . . . 307
Epidemic, Cost of . . . . . . . . 899
Esophagus, Dilatation of . . . . . 564
Examinations for License, Dividing the . . . . . . . . . . . . . 674
Expectorants and Cough Remedies . 731
Eye, Massage of . . . . . . . . . 155
Glasses . . . . . . . . . . . 341

FEVER, Enteric in Infancy . . 494
Treatment of Intermittent . . . . . . . . . . 333
Formalin for Eye Specimens . . . 650

Fractures, Treatment of Compound. 529
Frederick, C. C., M. D., Cardiac Embolism following Abdominal Section . . . . . . . . . . . . . 48
Frye, Maud J., M, D., Deformed Fetal Skull . . . . . . . . . . . 880

GASTROSTOMY in the Seventeenth Century . . . . . 395
Gall Stones, Removal of . . . . . 954
Georgia Medical Examiners . . . . 168
State Medical Examining Board . . . . . . . . . 740
Glycerine Injections for Induction of Labor . . . . . . . . . . . . . 661
Goitre, Symptomatology and Pathology of Exophthalmic . . . 793
Gold Combinations as Alteratives . 497
Gonorrhea, Abortive Treatment of . 655
in Women . . . . . . . . 660
in the Female . . . . . . 814
Gonorrheal Psychoses . . . . . . 809
Gram, Franklin C., M. D., A Healthy Community . . 258
Health Records . . . . . 346
Historical Sketch of the Medical Society of the County of Erie . . . . . 551
October Health in Buffalo. 415
November Vital Statistics . 496
Guaiacol as a Local Anesthetic . . 488

HAYD, HERMAN E., M. D., Large Ovarian Tumor, operation, recovery . . 37
Hysterectomy in Puerperal Sepsis . . . . . . . . 777
Hall, A. L., M. D., Fracture of Greater Tuberosity of Humerus . 379
Hauenstein, John, M. D., First uses of Chloroform and Ether in Buffalo . . . . . . . . . . . . . . . 689
Heads, A Study in . . . . . . . . 17
Headache, Treatment of . . . . . 369
Health Department of the Renaissance . . . . . . . . . . . . . 898
Heterophoria, Parallax Test for . . 153
Hill, Julian P., M. D., Sanitary Burial Casket . . . . . . . . . . 63
Himmelsbach, G. A., M. D., Enteric Fever Complicating Pregnancy 57
Home, Permanent for Medical Societies . . . . . . . . . . . . 825
Hopkins, H. R., M. D., Teaching of Physiology and Hygiene in the Public Schools . . . . . . . . . 289
Howell, H. Y., M. D., Nosocomial Hygiene . . . . . . . . . . . . . 320

PAGE.
Hubbell, Alvin A., M. D., Nuclear Ophthalmoplegia . . . . 39
Case of Otitic Brain Abscess . . . . . . . . . . . 782
Humerus, Fracture of Greater Tuberosity of . . . . . . . . . . . . 379
Huntley, Mary. M., M. D., Deformed Fetal Skull . . . . . . . . . . 880
Hydrocele . . . . . . . . . . . . . 489
Treatment of . . . . . . 489
of Labium Majus . . . . 818
Hydrozone in Purulent Otitis Media . . . . . . . . . . . . . . . 804
Hygiene, Nosocomial . . . . . . . . 320
Hymen Unruptured with Pregnancy . . . . . . . . . . . . . 248
Hysterectomy, Statistics of Vaginal. 816

IDIOTS' SKULLS . . . . . . . 810
Infantile Scurvy . . . . . . . 4
Inebriety, Epileptiform . . . . . . 811
Ingraham, Henry D., M. D., Ectopic Pregnancy . . . . . . . . . . 18
Insanity, Test for . . . . . . . . . 810
and Murder . . . . . . . 810
Intestinal Tract, Amebic Catarrh of . . . . . . . . . . . . . . 769

**Illustrations**:

A Broken Neck (Doyle). Figure 1 . . . . . . . . . . . . . 465
Figure 2 . . . . . . . . . . 466
Figure 3 . . . . . . . . . . 467
Andrews, Judson Boardman . . 101
A Study in Heads (Pohlman) . 19
Broad Ligament Pedicle (Tait), Figures 1, 2, 3, 4 . . 930
Buffalo Medical Journal, facsimile . . 66
Buffalo Fresh Air Mission Hospital, facing page . . . . . . 449
Buffalo General Hospital, 1858. 96
" " " 1880. 97
" " " 1896. 99
Buffalo, 1845 . . . . . . . . . 68
Buffalo, 1895, facing . . . . . 110
Buffalo State Hospital . . . . . 103
Buffalo Medical College, First . 78
Buffalo Medical College, Second 79
Buffalo Medical College, Third 83
Buffalo Woman's Hospital . . . 105
Cancer of the Breast (Mynter). 2
Carnegie Library Building, Pittsburg . . . . . . . . . 833
Cervical Tractor and Guide (Macbeth) . . . . . . . . . 820

PAGE.
Conformation of Medulla (Krauss) . . . . . . . . . 15
Davidson, A R.; Potter, Frank H.; Campbell, F. R. Facing page . . . . . . . . . . . 65
Deformed Fetal Skull (Frye and Huntley) . . . . 881
Dilatation of the Esophagus from Stricture . . . . . 567
Erie County Hospital . . . 107
Consumption Annex . . 109
Fillmore, Millard . . . 73
Flint, Austin; Hunt, Sanford B.; Minor, Julius F., facing page . . . . . . . . . . . 1
Fracture of Greater Tuberosity of Humerus (Hall). Figure 1 . . . . . . . . . . . . 381
Figure 2 . . . . . . . . . . . 383
Gay, Charles C. F. . . . . 87
Hamilton, Frank Hastings . . 70
Huxley, Thomas Henry . . . . 120
Irrigation of Peritoneal Cavity. 549
Mahlon Bainbridge Folwell . . 513
Matthews, James M. . . . . . 67
Michael, J Edwin . . . . . . 514
Myer, General Albert J. . . . . 77
Niagara University, Medical Department . . . . . . . . . 89
Nuclear Ophthalmoplegia (Hubbell) . . . . . . . . 41
Palmer, Edward Rush . . . . 123
Pasteur, Louis . . . . . . . . 355
Potter, Milton Grosvenor . . . 81
Pseudencephalic Monster (Conklin). Figure 1 . . . . 639
Figure 2 . . . . . . . . . . . 640
Retroflexion of the Gravid Uterus (Possaner). Figures 1, 2, 3 . . . . . . . . . . . 851
Rhinophyma (Wende) . . . 45
Rhinoscleroma (Wende). Figure 1 . . . . . . . . . . . . 643
Figure 2 . . . . . . . 645
Riverside Hospital, Operating Room (Randall) . . . . . . 873
Private Room . . . . . . . 875
Sitting Room and Double Ward . . . . . . . . . . . 877
Rochester, Thomas F. . . . . 75
Sanitary Burial Casket (Hill) . 63
Sisters of Charity Hospital . . 91
Second Sisters of Charity Hospital . . . . . . . . . . . . . 93
Spina Bifida (Park) . . . . . . 9
Stomach Washing (Pitkin) . . 229
Synchronous Amputations, (Daniels) pp. 50, 51, 52, 53, 55, 56
Syringe for Infiltration Anesthesia . . . . . . . . . . . . 527

PAGE.
Townsend, Franklin, Jr. . . . . 425
Trolley Ambulance, interior view . . . . . . . . . . 115
ready for use . . . . . 117
Tubercular Infection in Street Cars (Bissell) . . . . 47
White, James Platt . . . . . . 69
Wishard, Wm. N. . . . . . . . 127

**Instruments, New:**

Cervical Tractor and Guide . . 820
Syringe for Infiltration Anesthesia . . . . . . . . . . . . 527

JONES, WM. B., M. D., Vaginal Celiotomy . . . . . . . . 715

KRAUSS, WM. C., M. D., Histological Conformation of Medulla . . . . 12
Reflex Epilepsy . . . . 307
Symptomatology and Pathology of Exophthalmic Goitre . . . 793
Kidney, Removal of, and Ureter . . 956
Kuhlman, Helene, M. D., Case of Cerebral Softening . . . . . . . 864

LABOR, Bleeding from Varicose Veins in . . . . . . . . . . . 894

**Leading Articles:**

College Commencements and Alumni Association Meetings. 901
Economics of Municipal Charity . . . . . . . . 419
Electric Railway Ambulance . 114
Erie County Hospital . 174, 506, 909
Jubilee, Medical Dapartment University of Buffalo . . . . 743
May Meetings at Atlanta . . 970
Medical Society of the County of Erie, a Diamond Anniversary . . . . 587
Medical Society of the State of New York . . . . . . . . 665
Medical Department Niagara University . . . . . . . 823, 973
Noise Nuisance Again . . . . . 265
Object Lesson for Antivaccinists . . . . . . . . 909
Prof. Thomas H. Huxley . . . 119
Public Baths . . . . . . . . . . 269
Woman and the Bicycle . . 348, 908
Woman's Edition . . . . . . . 822
A. L. Koursh Co. . . . . . . . 206

PAGE.
**Literary Notes:**

Alumni Catalogue, University of Buffalo . . . . . . . . . . 607
Alvarenga Prize . . . . . . . . 447
American Year-Book of Medicine . . . . . . . . . . . . . 368
American Journal of Ophthalmology . . . . . . . . . . . 446
American Medical Review . . . 525
American Journal of Surgery and Gynecology . . . . . . . 526
Anesthesia, History of . . . . . 606
Archives of Pediatrics . . . . . 367
Atlantic Medical Weekly . . . 205
Blakiston, Son & Co. . . . . . . 687
Blakiston's Portrait Catalogue . 920
Buffalo Druggist . . . . . . . . 204
Buffalo Sunday News . . . . 765
Bulletin Harvard Medical Alumni Association . . . . . 526
Canada Medical Record. . 448, 767
Cleveland Journal of Medicine. 606
Cleveland Medical Gazette . . 448
Clinical Recorder . . . . . . . . 686
Coca and its Therapeutic Application . . . . . . . . . . . . 848
College of Physicians and Surgeons, Chicago . . . . . . 447
Complete Farrier and British Sportsman . . . . . . . . . . 446
Don'ts for Consumptives . . . 687
Drevet Manufacturing Co. . . 927
Dunglison's College and Clinical Record . . . . . . . . . 526
Hodgkin Prize paid . . . . . . 446
International Medical Annual . 524
Jenk's Prize . . . . . . . . . . 205
Journal of Medicine and Surgery . . . . . . . . . . . . . 766
Journal of Experimental Medicine . . . . . . . . . . . . . 607
Katherine Lauderdale . . . . . 605
Keil's Medical Directory . 206, 525
Ladies' Home Journal. 446, 526, 766
Langsdale's Lancet . . . . . . 686
Lays on Rays . . . . . . . . . 765
L'Union Medicale du Canada . 687
Medical and Surgical Register (Polk's) . . . . . . . . . . 448
Medical and Surgical Reporter. 205
Medical News . . . . . . . 526, 608
Medical Fortnightly . . . . . 606
Medical Mirror . . . . . . . . . 607
Medical Council . . . . . . 686
Modern Medical Science . . . 687
Monist for April . . . . . . . . 848
Nashville Journal of Medicine and Surgery . . . . . . . . . 205
National Medical Review . . . 926

PAGE.
New York Pharmacal Association. . . . . . . . . .
New York State Medical Reporter . . . . . . . . . . . . 686
Newer Remedies . . . . . . . 927
New Orleans Medical and Surgical Journal . . . . . . . . 446
Pediatrics . . . . . . . . . . . 448
Peoria Medical Record . . . . 847
Peoria Medical Journal . . . . 847
Physicians' Protective Association . . . . . . . . . . . 368
Popular Science . . . . . . 607
Protonuclein Clinical Records . 848
Quarterly Journal of Lunacy. . 447
Quarterly Medical Journal. . . 526
Report of Commissioner of Education . . . . . . . . 686
Reports received . . . . . . . 848
Rio Chemical Co. . . . . . . . 928
Scalpel . . . . . . . . 769
State Hospitals Bulletin . . . 686
Times and Register . . . . 608
Treatise on Surgery by American Authors . . . 927
Virginia Medical Semimonthly. 847
Weight Chart for Infants . . . 368
Weir's Index to the Medical Press . . . . . . . . 766, 927
Woman's Hospital, Surgical and Gynecological Clinic . . 927

Lungs, Bruises of . . . . . . . . . 20
Lupus Vulgaris, etc. . . . . . . . . 56
Lytle, Albert T, M. D., Work of State Examining Board . . . . 342

MACBETH, A. H., M. D., Cervical Tractor and Guide for Trachelorraphy . . . . . . . 820
Maine Medical Registration Board. 167
Maloney, F. W., M. D., Lupus Vulgaris, etc. . . . . . 561
Mann, Matthew D., M. D., Ectopic Gestation, Operation . . . . . 23
Mathews, J. M., M. D, Observations on Rectal Surgery . . . . . 129
Matzinger, Herman G., M. D., Origin and Nature of Pain . . . . . 137
Medical Journalism, Fifty years of . 65
Examining Board, Work of New York State . . . . 342
Examinations for U. S. Army . . . . . . . 974
Examining Boards, Relations of, to the State, Schools, and each other. 938
Examiners, Reciprocity between State Boards of. 345

PAGE.
Medical Expert Testimony . . . . 507
Society of the County of Erie, Historical Sketch of . . . . . . . . . 551
Students' Certificate . . . . 583
Legislation, Some needed . 595
Law, Violation of . . . . 670
Surgery . . . . . . . . . . 710
Teaching, Methods of . . 736
Training in New York . . 737
Department University of Buffalo, Establishment and Early Days of . . 773
Department, Niagara University, Establishment of . . . . . . . . . . . 780
Medulla, Histological Conformation of . . . . . . . . . . . . . 12
Membranes, Birth of Child without Rupture of. . . . . . . . . . 251
Memorial, Mahlon Bainbridge Folwell, M D. . . . . . . . . . . . 485
Menstruation, Cold Baths during . . 254
Messiah, A New . . . . . . . . . 422

**Miscellany:**

American Medical Association . 767
American Medical Publishers' Association . . . . . . . . . 767
Antikamnia Chemical Co. . . . 608
Army Medical Board . . . . . 288
Boislinière Prize Essay Fund . 768
Buffalo Antitoxic Company . . 366
Care of Persons found Unconscious on the Streets . . . 206
Charles Roome Parmele Co. . . 767
Craig Colony for Epileptics . . 364
Damages for Compulsory Vaccination . . . . . . . . . . . 528
Infiltration Anesthesia . . . . 526
King Thermometer Syringe . . 208
Medical Expert Testimony . . 687
Mellier Drug Co . . . . . . . 767
Palisades Manufacturing Co. Prizes . . . . . . . . . . 528
Parke, Davis & Co. . . . . . . . 608
Samuel D. Gross Prize . . . 528
St. Luke's Hospital, New York. 448
Ullenbruch, J. H . . . . 208
University Medical College of Kansas City . . . . . . . 767
Victor Koechl & Co . . . . . 688
Wilhelm Meyer Memorial . . . 928
Wm. F. Jenks Prize . . . . . . 928

**Medical College and Hospital Notes:**

Buffalo College of Dentistry, . . . . . . . . . . . 834, 977

PAGE.
Buffalo Hospital, Sisters of Charity . . . . . . . . . . 753
Children's Hospital . . . . . . 598
Erie County Hospital . . . . . 598
Erie Railway Hospital System . 834
Medical Department University of Buffalo . . . . . . 754
Post-Graduate Medical School and Hospital . . . . . 753
Riverside Hospital . . . . 754, 918
Rush Medical College . . . . 977

Microörganisms in the Healthy Nose . . . . . . . . . . . . 725
Microscope in Surgery . . . . . . 891
Midwifery, Digital Exploration in . 817
Milk, Certified . . . . . . . . . . 746
Danger of Pasteurised . . . 492
Miscarriage, Management of. . . . 817
Missouri Medical Colleges, Entrance Requirements . . . . . . 584
Moehlau, F. G., M. D., Gastrostomy in the Seventeenth Century . . . . 395
A Peculiar Case of Syphilitic Infection . . . . . 484
Monster, a Pseudencephalic . . . . 638
Moody, Mary Blair, M. D., College Life for Women . . . 854
Morris, Robert T., Clinical Lecture by . . . . . . . . . . . . 954
Mulford, Henry J., M. D., Obstructions of Upper Respiratory Tract in Children . . . . . . . 60
Mydriatics in refraction . . . . . . 394
Mynter, Herman, M D , Remarks on Cancer of the Breast . . . . . 1
Myopia, Convex Glasses in . . . . 340

NASAL TAMPON, Note on Posterior . . . . . . 397
National Confederation State Medical Examining Boards—Report of Committee on Organization . . . . . . 407
Program of . . . . . . 735
Guard Surgeons . . . . 509
Neck, A broken, recovery . . . . 464
Nephritis . . . . . . . . 656
Nervous Diseases, Reflex Causes of. 812
Nerve Suture . . . . . . . . . . . . 244
Neuralgia, Aconitine in . . . . . . 243
Neuralgia, Water in Treatment of . 733
Neuritis, Edematous Optic . . . . 336
New-born, High Mortality among. . 496
England, Practice Laws in . 585
Haven County Medical Association . . . . . . . . . 423
Mexico, Board of Medical Examiners . . . . . 412

PAGE.
Nuclein, Yeast in Hipjoint Disease. 896
Nursing as a Profession . . . . . . 347
Nussbaum Bill . . . . . . . . . . 748

OBSTETRIC BINDER . . . . 246
Obstetrics, Points in . . . . 247
Practical Observations in . . . . . . 894
Teaching of . . . 887
Ophthalmology, French Congress of . . . . . . . . . . . . . . . 974
Oregon Medical Practice Law . 413, 585
State Medical Examining Board . . . . . . . . . . 740
Ovary, Cyst of Left . . . . . . . 955

**Obituary:**

Battey, Robert . . . . . . . 426
Boislinière L. Ch. . . . . . . . 596
Carroll, Peter V. . . . . . 917
Comegys, C. G. . . . . . . 678
Conley, Arthur Victor . . . . 178
Corson, Hiram . . . . . . . . 750
Folwell, Mahlon Bainbridge . . 512
Hoyt, Horace . . . . . . . 975
Hunter, Alex. S. . . . . . . . . 678
Jaggard, W. W. . . . . . . . . 677
Jones, Joseph . . . . . . . . . 676
Keith, Thomas . . . . . . . . . 426
Mapes, James Jay . . . . . . 828
Michael, J. Edwin . . . . . . . 514
Norris, Basil . . . . . . 423
Palmer, Edward Rush . . . . 122
Pasteur, Louis . . . . . . . . 353
Reeves, James E. . . . . . . . 677
Retel, Michael . . . . . . . 917
Ripley, John H. . . . . . . . . 676
Roosevelt, James West . . . . 827
Schieffelin, Wm Henry . . . . 124
Seymour, Abby J. . . . . . . . 356
Stranahan, George. . . . . . . 124
Todd, Orrin . . . . . . . . 975
Townsend, Franklin, Jr. . . . 424
Weidman-Hynds, Frances. . . 597
Wheeler, George B . . . . . 918
Williams, Henry W. . . . . . . 177

Ohio State Medical Examining Board . . . . . . . . . . . 781
Old People, Care and Treatment of. 144
Ophthalmia, Purulent . . . . . . 337
Treatment of Granular . 804
Ophthalmoplegia, Nuclear . . . 39
Opium Addiction, Decadence of . . 392
Otitic Brain Abscess, Case of . . . 782
Ovarian Tumor, Large, Operation, Recovery . . . . . . . . . . . . 37

PAGE.

PARK, ROSWELL, M. D., Spina Bifida . . . . . . . . 8
Pain, Origin and Nature of . . . . 137
Palmer, George M., M. D., Recollections of a Country Practice . . 479
Paragraphs, Pilfered . . . 670
Paresis, Observations on General . 475
Parmenter, John, M. D., Bruises of the Lungs . . . . 209
Chloroform, Method of Administration, its Dangers and their Treatment . 695
Pedicle, Evolution of Surgical Treatment of the Broad Ligament . . . . . . . . . . . . . . 930
Peliosis, Relation of to the other forms of Idiopathic Purpura . . . 483
Pelvic Viscera, Lesions of . . . . 859
Pelvimeter, Use of . . . . . . . . 895
Pelvis, Female in Primitive Races . 895
Pennsylvania State Medical Examining Board . . . . . . . . . . 739
Peritoneal Cavity, Irrigation of . . 548
Peritonitis, Localised . . . . . . . 559

**Personal**:

Lewis, Daniel; Fowler, Geo. B. . . . . . . . . . 124
Jelks, Jas. T.; Satterlee, Richard H.; Love, I. N.; McMurtry, L. S.; Reed, Chas. A. L.; Lewis, Bransford; Grant, H. Y. . . . . . . . 125
Smith, Frederick W.; Murphy, John B; Hurd, A. W; Mulford, Henry J.; Hauenstein, John; Dorsett, Walter B.; Harrington, D. W.; Fowler, Joseph . . . . . . . . . . . 126
Harvey, L. F. . . . . . . . . . . 178
Mynter, Herman; Harrington, D. W.; Cott, George F.; Grant, H. Y.; Hurd, A. W.; Norton, Thos. H.; Hummel, A L.; Norbury, Frank P.; Meidenbauer, J. G.; Lytle, Albert T. . . . . 179
Smith, Eugene; Hall, Rufus B. . . . . . . . . . . . . . 204
Kidder, Walter H. . . . . . . 276
Dunn, Sherwood; Bissell, Wm. G.; Warren, John Collins; Thornbury, F. J.; Whipple, Electa B.; Moehlau, F. G. . 277
Gihon, Albert L. . 352
Wilcox, D G.; Eddy, J. L.; Ruffner, E. L; Holmes, C. R. . . . . . . . . . . 353
PAGE.
Putnam, James O.; Werder, X. O. . . . . . . . . . . . . . 427
Dowd, J. Henry; Long, Eli H.; Randall, Lillian C.; Daniels, J. H. . . . . . . . 428
Jewett, Chas. L. . . . . . . . . 448
Matzinger, H. G.; Grove, W. G.; Billings, John S; Harvey, L. F.; Engleman, Geo. J. . . . . . . . . . . . . . 511
Watson, William Perry . . . . 595
Abbott, A. C.; Rafter, Geo W.; Dowd, J. Henry; Brown, George L.; Smith, Chauncey, P. . . . . . . . . . . 596
Spencer, James G.; Novy, F. G. 675
Lewis, Daniel; Keiser, Max . . 676
Fisher, John C.; Reed, Chas. A. L. . . . . . . . . . 749
Brown, John Young, Jr.; Clark, Edward; Benedict, A. L; Mann, Frederick J.; Randall, Lillian C.; Griffith, Benjamin M. . . . . . . . . 750
Bennett, Arthur G.; Hayd, H. E.; Moehlau, F. G.; Storrs, Melancthon; Carstens, J. H.; Suiter, A Walter . . . 826
Rohé, Geo. H.; Dewey, Melvil . 827
Hutchinson, Woods . . . 835
Mosher, Eliza M; Dolly, Sarah R Adamson-; de Steiger, Miss A. I.; McMurtry, C. S; Reed, Chas. A. L. . . . . . . . . . . . . . . 916
Potter, Wm. Warren; Snow, Irving M.; Grosvenor, J. W.; Dagenais, Alphonse; Howe, Lucien . . . . . . . 917
McAdam, L. J.; Burrows, Lorenzo; Bacon, John E . 921
Frear, Jane N.; Preiss, Frederick; Preiss, Wm.; Finerty, J. J.; Putnam, J. W.; Pryor, John H. . . . . . . . 922
Mills, F. Hubbell; Forsyth, E. A. . . . . . . . . . . . . 974
Colton, A. J. . . . . . . . . . 975

Peterson, Frederick, M. D., Electricity in Nervous Diseases . 300
Physicians' Downtown Offices in New York . . . . . . . . . . 745
Physiology and Hygiene, Teaching of in Public Schools . . . . . . 289
Pitkin, John T., M. D., Stomach Washing . . . . . 228
Note on Nasal Tampon . . 397
Irrigation of Peritoneal Cavity . . . . . . . . . . 548
Placenta Previa . . . . . . . . . 801

PAGE.
Plaster of Paris, Application of . . 577
Pneumonia, Treatment of in Children . . . . . 897
Pohlman, Julius, M. D., A Study in Heads . . . . . . . . . 17
Possanner, Gabriele Baronin, M D , Retroflexion of the Gravid Uterus . . . . . . . . . . . 849
Potter, William Warren, M. D , Fifty Years of Medical Journalism, etc. . . . . . 65
Relations of Medical Examining Boards to the State Schools, etc. . . . 938
Practice, Recollections and Observation of a Country . . . 479
Pregnancy, Drugs Contraindicated During . . . . . . . . 306
Ectopic . . . . . . . . . 18
Enteric Fever Complicating . . . . 57
Obstinate Vomiting in . . 816
Operation . . . . . . . . 23

**Progress in Medical Science :**

Genito-Urinary and Syphilitic Diseases, by Byron Daggett, M. D. . . . . . 488, 653
Laryngology, by Henry J. Mulford, M. D. . . . . 401, 722
Medical Education and State Examinations, by William Warren Potter, M. D. . . . . . 165, 342, 407, 582, 734
Medicine, Pathology and Therapeutics . . . . . . . . 728
Neurology, by Wm. C. Krauss and Jas. W. Putnam . 241, 809
Obstetrics, Gynecology and Pelvic Surgery, by William Warren Potter, M. D. . . . . . . . . . . . . . 246, 657, 814
Obstetrics, Gynecology and Pelvic Surgery, by Electa B. Whipple, M. D. . . . 893
Pediatrics, by Maud J. Frye, M. D. . . . . . 159, 492, 896
Physiological Chemistry, by John H. Miller, Ph. D. . . . 156
Public Health, Hygiene, Bacteriology, by Ernest Wende, M. D. . 162, 258, 346, 415, 496, 812
Public Health and School Sanitation, by Ida C Bender, M. D., and Jane N. Frear, M. D. . . . . . . . . . . . . 898
Ophthalmology and Otology, by Alvin A. Hubbell, M. D., . . . . . . . 150, 336, 650, 804

PAGE.
Surgery, by John Parmenter, M. D. . . . . . . . . . . 575
Surgery, by Jane W. Carroll, M. D. . . . . . . . . . . . . 889
Puerperal Sepsis, Indications for Operation in . . . . . . 662
Hysterectomy in . . . 777
Purgatives, Hypodermatic . . . . 59
Putnam, James W. M. D., Criminal's Plea of Irresponsibility . . . 316
Pygophagus . . . . . . . . . . . . 492

RANDALL, LILLIAN CRAIG, M. D , Riverside Hospital . 872
Rectal Surgery, Observations on . . 129
Examinations of Pregnant Women . . 249
Regents, New York City Office . . 351
Department . . . 736
Remarkable Woman in Medicine . 910
Renner, W. Scott, M. D., Paroxysmal Sneezing . . . . . . . 31
Respiratory Tract Obstruction in Children . . . . . . . [illegible]0
Retina, Treatment of Detachment of . . . . . . . . . . . . . 150
Retinitis Albuminuria . . . . 152

**Reviews :**

Annual Report Illinois State Board of Health . . . . . . 285
Annual Report Pennsylvania State Board of Health 202
Ashurst : Encyclopedia of Surgery . . . . . . . . 602
Black : Urine in Health and Disease . . . . . . . . . . 362
Bracken : Materia Medica and Pharmacology . . . . . . . 924
Brothers : Infantile Mortality . 985
Brown : Diphtheria and its Associates . . . . . 754
Byford : Manual of Gynecology . . . . . . . . . . . . . 761
Claiborne : Examination of the Eye . . . . . . . . . . 835
DaCosta : Medical Diagnosis . 600
Dennis . A System of Surgery, Vol. I., 198 ; Vol. II., 279 ; Vol. III. . . . . . . . 682
Dennison : Exercise and Food for Pulmonary Invalids . . . 286
Dercum : Nervous Diseases . . 434
Dessar : Catarrhs and Colds . . 199
Duhring : Cutaneous Medicine. 518
Foster : Text-book of Physiology . . . . . . . . . . . . 521
Frothingham : Guide for Bacteriologists . . . . . . . . . 201

PAGE.
Fuller : Disorders of the Male Sexual Organs . . . . . . . 599
Fullerton : Obstetric Nursing . 925
Gautier : Special Pharmaceutic Formulary . . 284
Gillet : Formulaire des Medications Nouvelles . . . 989
Gould : American Year-Book of Medicine and Surgery . . 836
Gowers : Diseases of the Nervous System . . . . . . . . . 437
Grandin-Jarman : Pregnancy, Labor and the Puerperal State . . . . . . . 763
Gray : Nervous and Mental Diseases . . . . . 681
Green : Introduction to Pathology and Morbid Anatomy . . . . . . . . . . . . 683
Griffith : Care of the Baby . . 283
Haab : Atlas of Ophthalmoscopy . . . . . . . . . . . 984
Hare : Practical Therapeutics. 441
Hart : Diet in Sickness and Health . . . . . . . . . . 925
Hayem : Physical and Natural Therapeutics . . . . . . . . . 520
Helferich : Atlas of Traumatic Fractures and Dislocations . 986
Herrick : Medical Diagnosis . 519
Houston : Electricity in Electrotherapeutics . . . . . . . . 987
Huber ; Work in Histological Laboratory . . . 841
Index Catalogue, Library of the Surgeon-General's Office. 362
International Clinics : Vol. I. Sixth series. 1896 . . . . . 982
International Medical Annual . . . . . . . . 200, 840
Jackson : Skiascopy in Refraction . . . . . . . . . 359
Jacobi : Therapeutics of Infancy and Childhood . . . . 987
Jakob : Atlas of Normal and Pathological Nervous System 988
Jennings : Color Vision and Color Blindness . . . . . 843
Johns Hopkins Hospital Reports, Vol. IV, 362 ; Vol. V., 978 ; Vol. IV., No. 9 . . 990
Kaposi : Diseases of the Skin . 201
Keen-White : American Textbook of Surgery . . . . . . 838
King : Manual of Obstetrics . . 442
Knies : Diseases of the Eye . . 197
Leonard : Materia Medica and Therapeutics . . . . . . . . 286
Lockwood : Practice of Medicine . . . . . . . . . . . . . 924

PAGE.
Loomis : Text-book of Practical Medicine . . . . 522
Maisch : Manual of Materia Medica . . 361
Mansell-Moullin : Surgery . . 842
Martin : Diseases of the Stomach . . . . . 837
McFarland : Text-Book Upon Pathogenic Bacteria . . . . 983
Mitchell : Injuries of Nerves and Their Treatment . . . 284
Möller : Cod Liver Oil and Chemistry . . . . . . 440
Morris : Lectures on Appendicitis . . . . . . . . . . . 443
New York State Commission in Lunacy . . . . . . 982
Norris : Text-book of Obstetrics . . . . 603
Osler : Practice of Medicine. 679
Parvin : Science and Art of Obstetrics . . . . . 360
Purdy : Urinalysis and Urinary Diagnosis . . . . 841
Rochford : Neuroses of Childhood . . . . . 442
Rotch : Pediatrics . . . 922
Roth : Modern Materia Medica . . . . . . . . . . . . . . 520
Sachs : Nervous Diseases of Children , . . . 281
Sajous : Annual of the Universal Medical Sciences . . 439
Sanger : History of Prostitution . . . . . . . . . 361
Schimmelsbusch : Antiseptic Treatment of Wounds 284
Schweinitz: The Toxic Amblyopias . . . 979
Senn : Pathology and Treatment of Tumors . . . . . . . 601
Senn : Principles of Surgery . 845
Shibata : Obstetrical Pocket Phantom . . . . . . . . . . . 989
Shoemaker : Materia Medica and Therapeutics . . . . . . 844
Smith : Diseases of Infancy and Childhood . . . . . . . . 986
Simon ; Manual of Chemistry . 441
Snell : Eyesight and School Life . . . . . . . . . . 442
Spectacles and Eyeglasses . . 684
Starr : Diets for Infants and Children in Health and Disease . . . . . . . 990
Stedman : Twentieth Century Practice, Vol. III., 195 ; Vol. IV., 438 ; Vol. VI., 839 ; Vol. V. . . . . . . . . . . . . . . 981
Stimson : Manual of Operative Surgery . . . . . . . . . . . 844

PAGE.
Taylor: Venereal Diseases . . 763
Thompson: Practical Dietetics. 359
Transactions: American Association of Obstetricians and Gynecologists, 1894. 200
American Academy of Railway Surgeons . . . 285
Association of American Physicians . . . . . . . 521
American Gynecological Society . . . . . . 843
Colorado State Medical Society . . . . . . . . 988
Florida Medical Association 990
First Pan-American Medical Congress . . . . . . 985
Medical Association of Central New York . . . 285
Medical Society of the State of New York . . . 440
Oregon State Medical Society. . . . . . . . 986
Orleans Parish Medical Society . . . . . . . . 985
Southern Surgical and Gynecological Association, 1894 . . . 202
Texas State Medical Association . . . . . . . 991
Wisconsin State Medical Society . . . . . . . . 987
Treves; A System of Surgery, Vol. I. . . . . . . . . . 518
Tyson: Examination of Urine . 842
Visiting Lists: Lindsay & Blakiston, William Wood & Co., Lea Bros. & Co. . . . . 436
Walsham: Deformities of the Foot . . . . . . . . . . 283
Watson: Physicians and Surgeons of America . . . . . . 762
Woolsey: Hand-Book for Hospitals . . . . . . . . . . . . 983
Year Book of Treatment for 1896 . . . . . . . . . . . 684
Rickets, Maternal Factors in . . . 493
Ringing of Bells . . . . . . 667
Riverside Hospital . . . . . . . . 872
Rhinophyma, Case of . . . . . . 43
Rhinoscleroma, Case of . . . . . 642
Rhode Island, Effects of Medical Law in . . . . . . . . . . 741
Röntgen X Rays . . . . . . . . . . 668
in Destroying Bacilli . . . 747

SATTERLEE, R. H., M. D., Abuse and Use of Atropia in Eye Treatment . 149
Mydriatics in Refraction. 394

PAGE.
Scarlatina Angina, Bacteriological Study of . . . . . . . . . . . . . 883
Schauta's Clinic . . . . . . . 866
Schools, Medical Inspection in Boston . . . . . . . . . 899
Scientific Food Theory . . . . 272
Scurvy, Infantile . . . . 4
Sellstedt, L G., Dilatation of Esophagus (Translation) . 564
Serum Therapeutics, Preventive Inoculation of . . . . . . 609
Sterility of . . 815
Sexual Perversion and Crime . . 273
Skinner, Charles R. . . . . . . . . 351
Skull, Deformed Fetal . . . 880
Smith, Eugene A, M. D., Treatment of Compound Fractures . . 529
Sneezing, Paroxysmal . . . . 31
Snow, Sargent F., M D, Tuberculosis, Laryngeal, etc; Adirondacks as a Winter Health Resort . . . 385
Irving M., M. D, Symptomatology and Treatment of Infantile Diarrhea . . 449
Spleen, Removal of . . . . . . . 955
Sutures, Dogtail . . . . . . . . . 957

### Society Meetings:

American Association of Obstetricians and Gynecologists . . . . . . . 127, 356, 977
American Microscopical Society . . . . . . . . . . . 517, 833
American Laryngological, Rhinological and Otological . . . 834
American Medical Association. 919
American Gynecological Society . . . . . . 921
American Medical Publishers' Association . . . . . . . . . 751
American Academy of Medicine . . . . . . . . . . 752
American Laryngological Association . . . . . . . . 126
American Orthopedic Association . . . . . 127, 829, 920
American Public Health Association . . 279, 358, 752, 921, 976
Association of Military Surgeons of the United States . 833
Association of American Officers of the National Guard . 597
Atlanta Meetings . . . . . 828
Buffalo Ophthalmological Society . . . . . . . . 678
Buffalo Academy of Medicine . . . . . . . . . 277, 428
Cleveland Medical Society . . 358

PAGE.

Detroit Medical and Library Association . . . . . . . . 358
International Periodic Congress of Gynecology and Obstetrics . . . . . . . . . . 751
Iowa State Medical Society . . 753
Lake Erie Medical Society . . 976
Medical Association of Central New York . . . . . 279, 358
Medical Society of the State of New York . . . . . . . 357, 517
Medical Society of the County of Chautauqua . . . . . 515, 975
Medical Society of the County of Erie . . . . . . 516, 921, 965
Medico-Legal Society of Jersey City . . . . . . . . . . 358
Microscopical Club . . . . 516, 977
Mississippi Valley Medical Association . . . . . . 127, 278, 976
National Confederation of State Medical Examining Boards . . . . . 751, 919
New York State Medical Association . . . . . . . . . . . 920
Ohio State Pediatric Society . 832
Second Pan-American Medical Congress . . . . . . . 918, 976
Southern Surgical and Gynecological Association . . 357, 429
St Louis Academy of Medical and Surgical Sciences . . . . 517
Third International Congress of Dermatology . . . . 752
Tri-State Medical Society . . . . . . . . . . . 279, 430, 752
Western Association of Obstetricians and Gynecologists . . 517
Wisconsin State Medical Society . . . . . . . . . . . . . 921

Spina Bifida . . . . . . . . . . . 8
Spitting in Public Places . . . . . 593
Sputum, Points in Examination of . 260
Stanton, Wm., M. D., Antiseptics in Surgery . . . . . . . . 224
Placenta Previa . . . . . 801
Stanchfield Bill . . . . . . . . . . 673
State Hospital Managers, Change . 913
Stephenson, F. H., M. D., Observations on General Paresis . . . 457
Still-born, Resuscitation of . . 64
Street Cars, Tubercular Infection in . . . . . . . . . 46
Railway Company . . . . 350
Stomach, Contents of Healthy . . 157
Washing by a New Method. 228
Students of Medicine, Advice to . . 739
Syllabus, Medical . . . . . . . . . . 166
Syphilitic Infection, a Peculiar Case of . . . . . . . . . . . . 484

PAGE.

Syphilis, Treatment of at Aachen Springs . . . . . . . . . . . . . 490
Styptic, a New . . . . . . . . . . 577

**Society Proceedings :**

Buffalo Academy of Medicine . 886
Medical Society of the County of Chautauqua . . . . 172, 574
Medical Association of Central New York . . . . . . . . . . 398
Medical Society of the County of Erie . . . . . . 485, 570, 663
National Confederation State Medical Examining Boards . 950

TACHYCARDIA at the Menopause . . . . . . . . . . . 657
Tait, Lawson, Evolution of Surgical Treatment of Broad Ligament Pedicle . . . . . . . . . . . 930
Then and Now, 1845—1895 . . . . 65
Toms, S. W. S , M. D , Appendicitis . . . . . . . . . . . 537
Tonsil, Hypertrophy of the Lingual 878
Topics of the Month . 176, 269, 350, 421, 507, 589, 667, 745, 824, 912, 974
Torticollis . . . . . . . . 245
Trichiasis. Operative Treatment of 651
Trional. Experiments with . . . 328
Tuberculosis, Aural, Nasal and Laryngeal . . . . . . 385
Surgical Treatment of Laryngeal . . . . . . 401
Peritoneal . . . . . . . . . 579
and Genital Infection . . . 803
Effects of on Dairy Cattle . 824
Typhoid Fever, Epidemic at Elmira . . . . . . . . . . . 745
in Children . . . . . . . . 897

URACHUS, Cystic . . . . . . 869
Uterus, Lateral Displacements of . . . . . . . 660
Retroflexion of Gravid . 849
Vaginal Extirpation of. . 815
Vaginal Fixation of . . . 893
Urinary Tract, Flooding of the . . 231
Suppression, a Case of . . 489
Tract, Infection of . . . . 490
Examinations . . . . . 654
Urine, Import of Casts in . . . . . 654
Test for Pus in . . . . . . 655
Ulyptol . . . . . . . . . . . . . 47

Page.

VAGINA, Absorptive Power of . 252
Round Ulceration of . 256
Vaginismus, Pathology of . . . . . 255
Vaginal Secretions, Gonococci in . . 256
Celiotomy . . . . . . 715
Vandenbergh, Bina Potter-, M. D., Lesions of Pelvic Viscera . . . . 859
Vitapaths in Cincinnati . . . . . 505
Vivisection in the District of Columbia . . . . . . . . . . . 915
Vomiting of Pregnancy . . . . . . 249
Vulva, Impregnation from Semen Deposited on . . . . . . . . . . 257

WATER METERS . . . . . . 421
Supply of Buffalo.591. 675
Washington State Medical Examining Board . . . . . . . . . 742
Wende, Ernest, M D., A Case of Rhinophyma . . . . . . 43
G. W., M. D, Case of Rhinoscleroma . . . . . . . 642

Page

Whipple, Electa B., M.D., Schauta's Clinic . . . . . . . . . . . 866
Winslow, Dr. Forbes and Mrs. Maybrick . . . . . . . . . . . 271
Women Physicians in the State Hospitals . . . . . . . 674
Employment of in Mercantile Establishments . . . 913
Enrolled as Students . . . 915
Graduates in Buffalo . . . 912
London School of Medicine for . . . . . . . . . 914
New Hospital for in London . . . . . . . . . . 914
Notes about . . . . . . 916
Wyckoff, C C., M. D., Establishment and Early days of University of Buffalo . . . . . . . . . 773

YOUNG, A. A., M. D., Amebic Catarrh of the Intestinal Tract . . . . . . . . . . . . 769

